# Case Report: Marked bilateral optic disc edema as the presenting manifestation of thyroid eye disease with dysthyroid optic neuropathy

**DOI:** 10.3389/fopht.2026.1870279

**Published:** 2026-07-27

**Authors:** Isabella V. Y. Gomes, Sara Krachmalnick, Christine Ryu, Leanne Stunkel

**Affiliations:** 1 Washington University School of Medicine, St. Louis, MO, United States; 2 John F. Hardesty, MD Department of Ophthalmology & Visual Sciences, Washington University School of Medicine, St. Louis, MO, United States; 3 Department of Neurology, Washington University School of Medicine, St. Louis, MO, United States

**Keywords:** diagnosis, dysthyroid optic neuropathy, Graves’ disease, optic disc edema, thyroid eye disease

## Abstract

**Purpose:**

The aim of this study was to report an atypical presentation of dysthyroid optic neuropathy (DON) characterized by severe bilateral optic disc edema mimicking papilledema.

**Methods:**

A 71-year-old man with hypothyroidism presented with 6 months of progressive bilateral vision loss and Frisén grade 4–5 optic disc edema. Comprehensive neuro-ophthalmic evaluation, orbital imaging, and laboratory testing were performed to establish the diagnosis.

**Results:**

Despite presenting with severe optic disc edema typically associated with papilledema, imaging revealed Type 2 thyroid eye disease with apical muscular crowding exceeding 67% and elevated thyroid-stimulating immunoglobulin. Normal lumbar puncture opening pressure excluded elevated intracranial pressure. Following treatment with high-dose intravenous methylprednisolone and staged bilateral three-wall orbital decompression, visual acuity improved from 20/30 to 20/20 bilaterally, with complete resolution of disc edema and visual field defects at 3 months follow-up.

**Conclusions:**

Severe optic disc edema is an uncommon but important presenting feature of DON that may mimic papilledema. This case emphasizes the critical importance of considering thyroid eye disease in the differential diagnosis of bilateral optic disc edema, even in the absence of prominent orbital signs, and demonstrates excellent visual outcomes with prompt corticosteroid therapy and surgical decompression.

## Introduction

Thyroid eye disease (TED) is an autoimmune inflammatory disorder, most commonly associated with Graves’ disease, in which autoantibodies against thyrotropin (TSH) receptors and adjacent insulin-like growth factor-1 (IGF-1) receptors on orbital fibroblasts trigger adipogenesis, T-lymphocyte activation, cytokine release, inflammation, and glycosaminoglycan deposition in orbital fat and muscle (1). These immune-mediated changes increase orbital volume and pressure, manifesting as proptosis, lid retraction, extraocular muscle dysfunction, and, in severe cases, compressive optic neuropathy (1).

Dysthyroid optic neuropathy (DON) is a vision-threatening complication occurring in 5%–8% of patients with TED, typically due to compression of the optic nerve at the orbital apex by enlarged extraocular muscles, which disrupts axoplasmic flow (1). Risk factors for DON include older age, male sex, smoking, and diabetes (1). Optic disc edema may be seen in 20%–50% of DON cases, but is typically mild (2, 3). Severe optic disc edema, such as grade 4 or grade 5 on the Frisén scale, typically correlating to elevated retinal nerve fiber layer (RNFL) greater than 200 μm is extremely unusual (4). Of note, proptosis is not universally present in patients with optic neuropathy due to TED (1).We present an unusual case of TED with severe bilateral optic disc edema (Frisén grade 4–5) as the presenting manifestation, highlighting the diagnostic challenges and importance of early recognition and intervention in this vision-threatening presentation.

## Materials and methods

A retrospective chart review was performed for a patient presenting with bilateral optic disc edema subsequently diagnosed with DON. Clinical data including visual acuity, color vision testing, visual fields, fundus examination, optical coherence tomography (OCT), orbital imaging, and laboratory studies were collected. Treatment details and outcomes were documented. Institutional review board approval was obtained, and the study adhered to the tenets of the Declaration of Helsinki.

### Consent to participate

Written informed consent for participation in this study was obtained from the patient.

### Consent to publish

Written informed consent to publish clinical details and images has been obtained from the patient. All efforts to anonymize the individual have been taken, including the removal of identifiable features from clinical photographs.

## Case report

A 71-year-old man was referred for evaluation of bilateral [oculus uterque (OU)] optic disc edema. He reported 6 months of progressively worsening bilateral blurred vision, right eye [oculus dexter (OD)] worse than left eye [oculus sinister (OS)], accompanied by eyelid swelling, mucous discharge, and ocular irritation. He endorsed several weeks of intermittent oblique binocular diplopia and mild frontal headaches with a sensation of pressure behind both eyes. He denied transient visual obscurations or pulsatile tinnitus. Past medical history included migraines, hypothyroidism (diagnosed 4 years earlier following radioactive iodine ablation for Graves’ hyperthyroidism, currently treated with levothyroxine), heart failure, type 2 diabetes mellitus, hyperlipidemia, hypertension, gout, and benign prostatic hyperplasia. He was a former smoker who had quit 14 years prior after smoking 2 packs per day for 11 years (22 pack-years).

**Figure 1:**
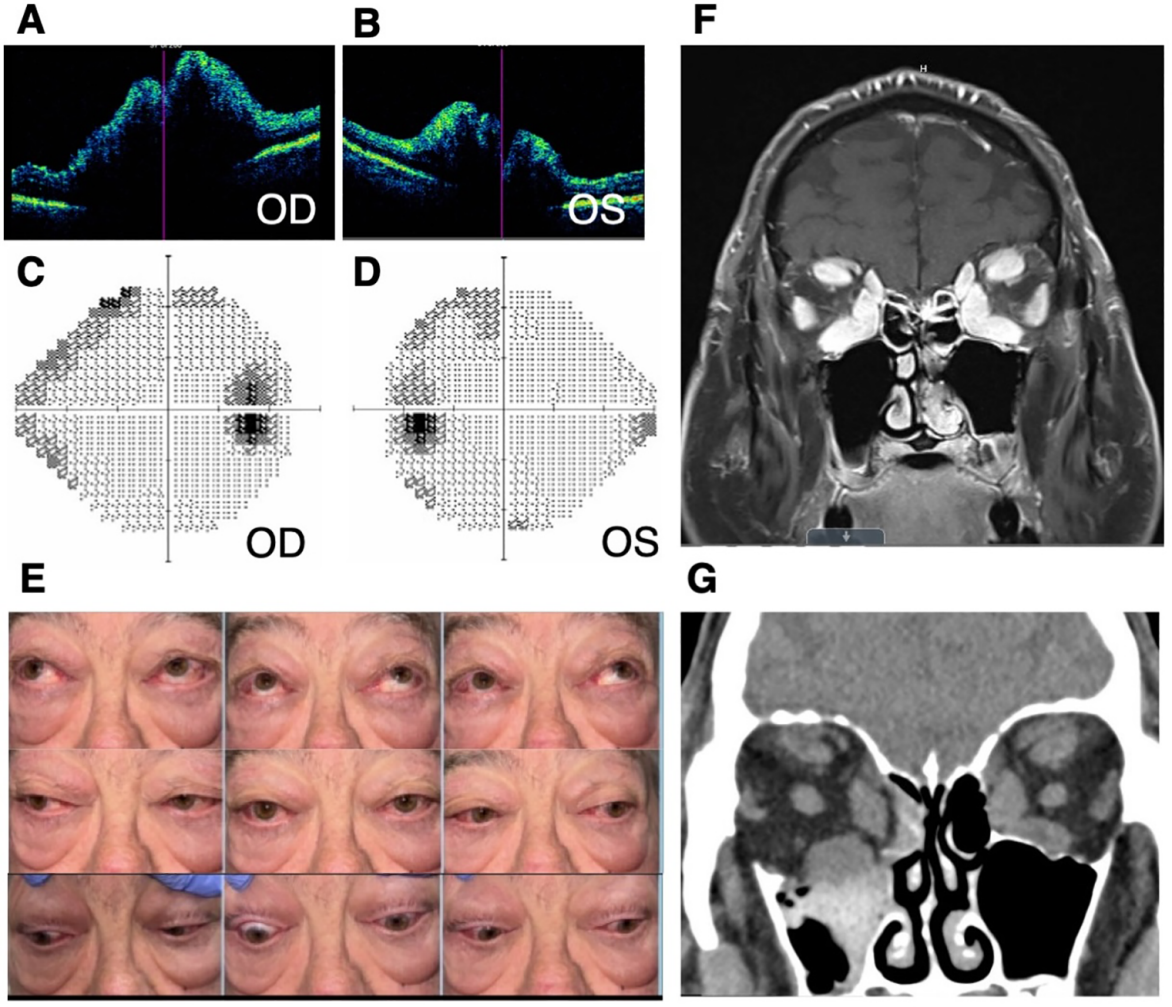
Composite clinical and radiographic findings in dysthyroid optic neuropathy (DON). Fundus photography was not obtained; optic disc edema was assessed clinically on funduscopic examination. **(A, B)** Optical coherence tomography demonstrating marked retinal nerve fiber layer elevation measuring 287 μm OD and 302 μm OS. **(C, D)** Humphrey visual field testing 24–2 demonstrating bilateral enlarged blind spots and early visual field constriction in the right eye (OD) and left eye (OS). **(E)** External and nine-gaze photographs illustrating conjunctival injection, chemosis, periorbital swelling with festoons, lower lid dermatochalasis, and diffuse extraocular motility deficits, particularly in abduction and right eye elevation. **(F)** Coronal T1-weighted post-contrast magnetic resonance imaging obtained at initial presentation showing severe extraocular muscle enlargement with contrast enhancement and orbital apex crowding. **(G)** Post-operative coronal computed tomography of the orbits without contrast approximately 1 week after right orbital decompression demonstrating successful decompression of the right orbit.

On examination, best-corrected visual acuity was 20/30 OU with mild color vision deficits (12/14 OD; 11/14 OS on Ishihara plates). There was no relative afferent pupillary defect. Fundoscopy demonstrated grade 4–5 optic disc edema with obscuration of vessels and peripapillary hemorrhages OU. Fundus photography was not obtained. OCT showed marked retinal nerve fiber layer (RNFL) elevation measuring 287 μm OD and 302 μm OS (**Figures 1A, B**). Key clinical and diagnostic findings are summarized in **Table 1**. Visual field testing revealed bilateral blind spot enlargement and early visual field constriction (**Figures 1C, D**). External examination revealed conjunctival injection, chemosis, periorbital swelling with festoons, and lower lid dermatochalasis (**Figure 1E**). Although some periorbital edema was present, the degree of clinical congestion was relatively mild compared to the severity of the optic nerve involvement. Hertel exophthalmometry demonstrated asymmetric proptosis measuring 24 mm OD and 26 mm OS. Intraocular pressure was normal in both eyes at 14 mmHg OD and 13 mmHg OS (normal range: 10–21 mmHg). Ocular motility demonstrated bilateral abduction deficits and a right elevation deficit (**Figure 1E**). Alternate cover testing revealed an incomitant left hyperphoria.

**Table 1:** Summary of key clinical and diagnostic findings.

Parameter	Initial presentation	3-month follow-up
Visual acuity (BCVA)	20/30 OU	20/20 OU
Color vision (Ishihara plates)	12/14 OD; 11/14 OS	Normal bilaterally
Optic disc appearance	Frisén grade 4–5 edema OU	Resolution of edema
OCT RNFL thickness	287 μm OD; 302 μm OS	86 μm OD; 91 μm OS
Visual field	Blind spot enlargement, early constriction	Substantially improved
Hertel exophthalmometry	24 mm OD; 26 mm OS	19 mm OU
TSI level	4.21 IU/L (elevated)	Not repeated
MRI muscular index	>67% at orbital apex	Improved post-decompression

BCVA, best-corrected visual acuity; OU, oculus uterque (both eyes); OD, ocular dexter (right eye); OS, oculus sinister (left eye); OCT, optical coherence tomography; RNFL, retinal nerve fiber layer; TSI, thyroid-stimulating immunoglobulin; MRI, magnetic resonance imaging.

Magnetic resonance imaging (MRI) demonstrated marked, diffuse enlargement and contrast enhancement of the bilateral extraocular muscles consistent with active inflammation and edema, exhibiting the characteristic tendon-sparing pattern typical of TED (**Figure 1F**), extending to the to the orbital apex, resulting in significant crowding and apparent compression of the bilateral optic nerves. This calculated muscular index exceeded 67% at the orbital apex bilaterally, a threshold highly suggestive of compressive optic neuropathy from TED (5). The optic nerve sheath complexes demonstrated increased T2 signal, further indicative of compressive and inflammatory changes, while the orbital fat compartments showed minimal involvement (**Figure 1F**).

At the time of imaging, the pronounced degree of bilateral apical crowding with optic nerve compression, in the context of severe disc edema and relatively preserved central visual function, raised suspicion for compressive optic neuropathy secondary to TED. However, given the atypical severity of the optic disc edema, alternative etiologies including papilledema from elevated intracranial pressure and orbital or systemic infiltrative processes such as inflammatory disease or malignancy were considered. Additional diagnostic evaluation included laboratory studies. Inflammatory markers were normal (ESR 11 mm/h, CRP <5.0 mg/L), effectively excluding giant cell arteritis. Thyroid function tests indicated a euthyroid status with normal TSH (1.11 mIU/L) and free T4 (0.97 ng/dL); however, thyroid-stimulating immunoglobulin was elevated (TSI 4.21 IU/L, reference range: <0.55 IU/L). Additional workup including ANA, ACE, ANCA, IgG4, HIV, treponemal antibody, RPR, tuberculosis testing, MOG, and NMO antibodies was unremarkable. Lumbar puncture showed normal opening pressure (15 cm H_2_O), normal CSF glucose (49 mg/dL), mildly elevated nucleated cells (10 cells/μL), normal CSF protein (47 mg/dL), and negative cytology and VDRL. These findings excluded increased intracranial pressure as well as infectious and neoplastic causes.

Given the otherwise negative workup, the diagnosis of compressive optic neuropathy from TED was made. The patient was initially treated with high-dose intravenous methylprednisolone (500 mg twice daily for 3 days) followed by an oral prednisone taper. Because of the severity of optic nerve compression, a staged bilateral three-wall orbital decompression was done for maximal decompression—first on the right orbit, followed by the left orbit approximately 4 weeks later. The surgical approach included a transcaruncular medial wall decompression and decompression of the floor and lateral wall through transconjunctival and lateral canthal incisions. High-dose intravenous corticosteroids were administered prior to surgical intervention to reduce orbital inflammation, decrease tissue edema, and facilitate surgical visualization and tissue manipulation (6).

At 3 months after bilateral decompression surgery, visual acuity had improved to 20/20 bilaterally, color vision normalized, visual field defects substantially improved, and funduscopic examination demonstrated resolution of the optic disc edema with OCT RNFL measuring 85 μm OD and 94 μm OS. Post-operative computed tomography at approximately 1 week after decompression surgery in the right eye demonstrated successful orbital apex decompression (**Figure 1G**). Approximately 10 weeks after right eye surgery and 6 weeks after left eye surgery, Hertel exophthalmometry had improved to 19 mm OU.

Following orbital decompression, the patient exhibited significant postoperative worsening of the restrictive strabismus, with a 40 prism diopter esotropia and 20 prism diopter right hypotropia in primary position attributable to fibrotic extraocular muscle changes. Strabismus surgery was subsequently performed, consisting of bilateral medial rectus recessions and right inferior rectus recession, to address the new-onset restrictive esotropia and right-sided hypotropia. The patient reported symptomatic improvement after the initial procedure. Further strabismus surgery is anticipated to address residual misalignment.

## Discussion

This case highlights an uncommon presentation of DON with profound bilateral optic disc edema (Frisén grade 4–5) at onset. While optic disc edema may be seen in 20%–50% of DON cases, it is typically mild (2, 3). In most cases, DON is associated with reduced RNFL thickness, typically ranging 63.5–111.4 μm (median 99.7 μm) (7). In contrast, severe papilledema (the most plausible alternative diagnosis in this case) is characterized by markedly elevated RNFL thickness, often exceeding 200–300 μm, with studies reporting mean values up to 337 ± 102 µm (8). Our patient’s RNFL measurements of 287 μm OD and 302 μm OS placed this case in the range typically associated with papilledema, creating a significant diagnostic challenge.

The pathophysiology of disc edema in DON differs fundamentally from that of papilledema. In DON, edema results from mechanical compression causing impaired axoplasmic flow and accumulation at the optic nerve head, plus venous congestion and direct inflammatory effects (4), whereas papilledema arises from raised intracranial pressure, transmitted along the optic nerve sheath.

In our case, MRI findings confirmed Type 2 (myogenic) TED, characterized by marked extraocular muscle enlargement and muscular indices exceeding 67% at orbital apex (5). This subtype is most frequently associated with compressive optic neuropathy due to apical crowding and optic nerve compression. Type 1 (lipogenic) TED is primarily driven by fat expansion and levator muscle scarring (9). Accordingly, a three-wall decompression was selected as the optimal intervention to relieve the severe apical compression.

Several large series have demonstrated favorable outcomes with appropriate treatment of DON. In a series of 40 patients (65 eyes) treated with intravenous steroids, radiotherapy, and orbital decompression, all patients showed improvement in visual acuity by two lines or more. (10) Another study of 33 patients who underwent orbital decompression for DON reported long-term preservation of visual acuity in 94% of patients. (11) More recently, a tertiary center study of 24 patients with DON demonstrated significant vision improvement with combined treatment approaches, with average improvement of two Snellen chart lines and resolution of relative afferent pupillary defect in all patients at final follow-up. (12) These larger series confirm that DON, when promptly recognized and treated, generally has favorable visual outcomes, as demonstrated in our patient who achieved 20/20 vision bilaterally.

Management of DON continues to evolve with targeted therapies such as teprotumumab, an anti–IGF 1 receptor monoclonal antibody, which has shown rapid improvement in DON, including steroid- and surgery-refractory cases, with most patients experiencing significant gains in visual acuity after early infusions. (13) A recent study of teprotumumab in steroid and surgery-refractory DON demonstrated recovery in 87.5% of affected eyes. (14) Other biologic agents, such as rituximab and tocilizumab, which deplete B cells and block interleukin-6 (IL-6) signaling, respectively, may offer alternatives or adjuncts to surgical decompression, particularly with progressive or refractory cases of DON (15).

Our patient’s history of radioactive iodine treatment for Graves’ hyperthyroidism is noteworthy, as radioiodine therapy is associated with increased risk of TED progression and DON development. (2) The subsequent hypothyroidism requiring levothyroxine replacement is a common sequela of ablative therapy. The development of severe DON 4 years after radioiodine treatment underscores the importance of long-term ophthalmologic surveillance in patients with treated Graves’ disease.

This case illustrates several important clinical teaching points. First, severe optic disc edema can be the presenting feature of DON and should not exclude this diagnosis. Second, DON should be considered in the differential diagnosis of bilateral optic disc edema, even when orbital congestion appears relatively mild. Third, the presence of previous thyroid disease, elevated thyroid autoantibodies, and characteristic imaging findings of apical muscle enlargement with tendon sparing are critical diagnostic features. Finally, prompt treatment with high-dose corticosteroids and appropriate surgical decompression can result in excellent visual outcomes, emphasizing the importance of early recognition and intervention.

## Conclusion

This case highlights a rare presentation of DON with severe bilateral optic disc edema mimicking papilledema. Prompt treatment with high-dose corticosteroids and bilateral three-wall orbital decompression led to full visual recovery and resolution of disc edema. This case underscores the importance of maintaining a high index of suspicion for DON in patients presenting with bilateral optic disc edema, even in the absence of prominent orbital congestion, and demonstrates the potential for TED-related optic neuropathy to present with severe disc edema.

## Patient perspective

“When my vision kept getting worse over several months, I was really worried. I’m incredibly grateful that my doctors acted quickly with the steroids and surgery—my vision is back to 20/20 now, which I never thought would be possible. While I still need more surgery for the double vision, I’m relieved my sight was saved”.

## Data Availability

The original contributions presented in the study are included in the article/**Supplementary Material**. Further inquiries can be directed to the corresponding author.

## Supplementary material

Supplementary Figure 1 Optical coherence tomography of the optic nerves 3 months post-operatively showed complete resolution of the optic disc edema.

Supplementary Figure 2 Optical coherence tomography of the macula from the time of patient’s initial presentation, showing retinal folds due to the degree of papilledema in both eyes, but no other retinal pathology.
